# Reducing emergency admissions and length of stay by introducing emergency surgical ambulatory service

**DOI:** 10.1016/j.amsu.2019.05.004

**Published:** 2019-05-18

**Authors:** M.A. Kazem, C. Hopley, D.J. Corless

**Affiliations:** a Surgery and Cancer Division, Leighton Hospital, Middlewich Road, Crewe, CW1 4QJ, UK; b Transformation Team, Leighton Hospital, Middlewich Road, Crewe, CW1 4QJ, UK

**Keywords:** Emergency, Surgical, Ambulatory, Care

## Abstract

**Background:**

Emergency surgical ambulatory care provides safe and effective assessment of acute surgical referrals, in addition to reducing the pressures on hospital beds.

Our aim was to look at the effect of opening a surgical ambulatory care unit (SACU) and a dedicated surgeon for the unit on length of stay and same day discharge for emergency referrals.

**Methods:**

Data was collected prospectively and updated daily to include all referrals to SACU. Historical data was retrieved to compare the effect of introduction of SACU and dedicated surgeon on same day discharge and length of stay.

**Results:**

Three groups of patients were identified: pre-SACU, SACU and SACU with dedicated surgeon. There was 104.5% percentage increase in same day discharge rate for emergency GP referrals (22% pre-SACU to 45% in the dedicated surgeon group). Similarly, same day discharge for all emergency referrals increased from 17% pre-SACU to 29% in the dedicated surgeon group.

There was 25.88 h reduction in the mean length of stay for emergency GP admissions (92.95 h pre-SACU to 67.07 h in the dedicated surgeon group). In pre-SACU group mean length of stay for all emergency admissions was 125 h, this dropped to 107.09 h in the dedicated surgeon group. This resulted in 102 hospital bed stays saved every month since the opening of SACU.

**Conclusions:**

Establishing an emergency surgical ambulatory service has reduced length of stay and saved significant hospital bed stays. This effect was enhanced by having a dedicated senior surgeon providing early input and decision making.

## Highlights

• Surgical ambulatory care reduced length of stay for emergency surgical admissions and increased same day discharge.
• Allocating a dedicated senior surgeon to the ambulatory service significantly improved the positive impact of this service.
• For the ambulatory unit to provide its intended outcomes it needs to work from a protected clinical area.
• A dedicated team, easy access to investigations and support from senior management are essential for SACU success.

## 1 Introduction

The increased demand on hospital beds, associated with an increase in ED attendances, requires a change in practice and approach. Data collected by NHS Providers during the winter months of 2016/7 showed emergency admissions consistently greater than 87,000 per week, with the number of escalation beds opened in one week exceeding 31,000 [1].

The aim of ambulatory emergency care (AEC)/same day emergency care (SDEC) is to deliver same day emergency care for patients who are considered for emergency admission [2]. Over the last five to ten years there has been an increase in the use of this concept in medicine and emergency departments, and more recently in general surgery [3]. The importance of AEC has already been recognized by NHS England, with the A&E Plan (2016) requiring all acute trusts to deliver AEC services [3].

If AEC is to deliver the intended outcomes, it is crucial to apply certain principles, which will facilitate early assessment and decision-making (3,4). Early senior review, and clear exclusion criteria are very important to ensure appropriate patients are referred to an AEC unit and a clear management plan is put in place as soon as possible [3,4].

It has been recognized that front door input from a senior surgeon will be able, in an appropriate setting, to reduce emergency admissions by 20–30% [5]. In addition, AEC shares a lot of day case surgery principles [4], which makes implementing it in the surgical setting feel intuitive. Over the last few years, there have been different approaches to surgical AEC, with many hospitals reporting how they have managed to build their units and publishing their outcomes [[6], [7], [8], [9], [10]]. These experiences all shared the same aspiration for early assessment and early discharge of emergency surgical patients, facilitated by early input from a senior surgeon at the front door.

We developed an emergency surgical ambulatory care unit with the aim to provide early assessment and management for patients who were referred by primary care providers to the surgical team as an emergency admission.

### 1.1 The unit

The Surgical Ambulatory Care Unit (SACU) was launched in September 2016 with clear objectives to achieve (box1). It opened Monday to Friday, 12:00 to 20:00, staffed by one Advanced Nurse Practitioner (ANP), a Staff nurse and two health care assistants. At the beginning the unit was covered by the on-call team with active input from the ANPs to help with initial assessment and triage. Shortly after that it was clear that the unit was not meeting its targets, and reassessment of how the unit was functioning was required.

Box 1 **SACU Objectives and KPI's:**

1. To reduce number of emergency surgery admissions:
  1a 70% of patients to be discharged home the same day
  1b 30% of surgical take Monday to Friday to go to SACU
  1c 30-day readmission rates for patients discharged directly from SACU to be less than 8.5%
  1d 7-day readmission rates for patients discharged directly from SACU to be less than 4%
2. To reduce the number of overnight stays of emergency patients:
  2a LOS on SACU to remain under 8 h
  2b LOS of emergency surgery patients to be reduced
  2c To reduce surgical bed days to achieve a bed day saving for emergency general surgery patients
  3. All patients on SACU to receive a clinical review by a senior decision maker:
  3a 85% of all patients attending SACU to have their first clinical review by a senior decision maker within 4 h
  3b 75% of all patients attending SACU to have their first clinical review by a senior decision maker within 2 h
4. To improve patient experience:
  4a 90% of SACU patients likely to recommend the surgical ambulatory care unit

Alt-text: Box 1

**Fig. 1:**
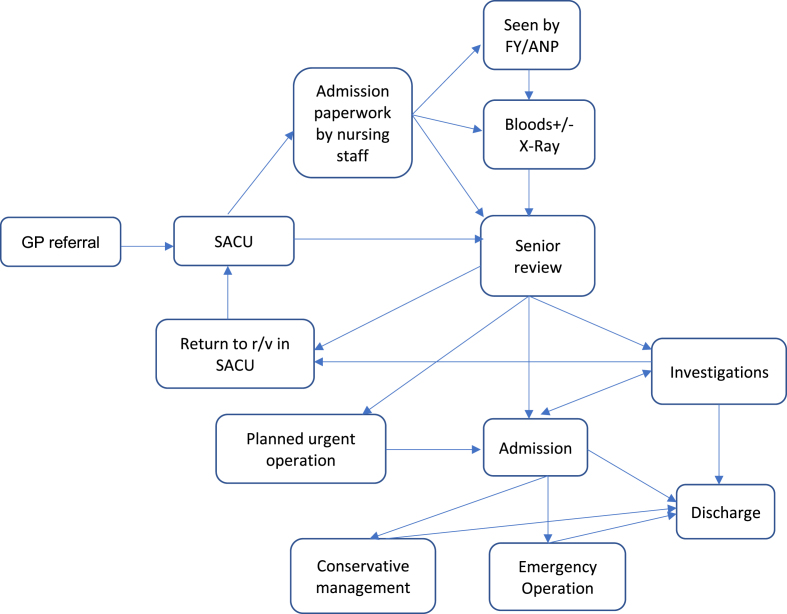
Flow diagram of SACU before introducing dedicated senior surgeon.

**Fig. 2:**
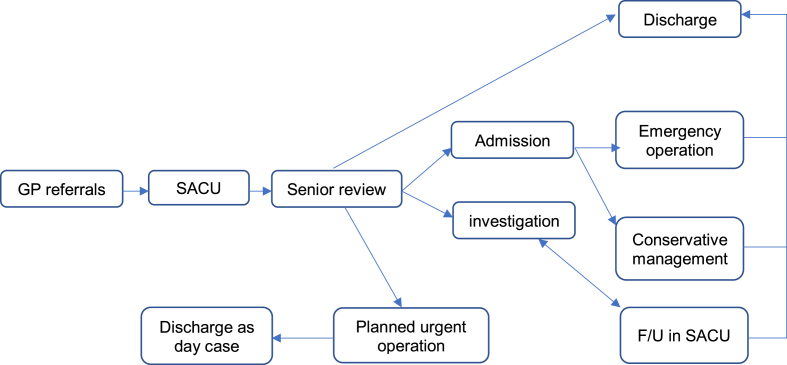
Ideal Flow diagram for SACU referrals.

A flow diagram of what happened to SACU admissions was performed (Fig. 1). It was clear that the main problem was lack of early input from a senior decision-maker. This, in addition to the lack of clinical leadership of the unit resulted in failure to meet targets. We discussed an ideal process for the unit (Fig. 2), and two senior surgeons, joined by a third surgeon later, showed interest in running the unit and providing the senior input needed to deliver the service five days a week.

In September 2017, SACU started offering early senior input Monday to Friday 13:30 to 17:30, through dedicated time slots similar to an outpatient setting. Patients have base line blood tests and simple X-rays done prior to being seen by the senior decision maker. These slots were also used to facilitate ambulatory scans and reassessment of patients discharged from previous SACU days or emergency inpatient episodes. The ANPs and the nurse in charge of the unit manage these slots on daily basis. A clear inclusion and exclusion criteria (box 2) were set to enable this process and ensure no hold ups occur in the unit.

Box 2 **SACU conditions list:**

Condition appropriate for SACU:

• RUQ pain
• LIF pain
• RIF pain
• Abscesses
• Stable PR bleeding
• Returning patients for investigations results

Conditions not appropriate for SACU:

• Patients who are not deemed ambulatory by their GP
• Bowel obstruction
• Patients with NEWS >3 or NEWS = 3 in one single parameter
• GP thinks patient cannot wait for the allocated time slot in SACU

Alt-text: Box 2

The unit has access to two dedicated Ultrasound scan slots per day and ability to book ambulatory CT and MRI scans, in order to facilitate early discharge and decision-making. Following any clinical contact with the patient, a discharge letter is produced and sent to both the GP and the patient.

Here we report on our outcomes since opening SACU, with the aim to demonstrate the effect of SACU and dedicated senior surgeon input on same day discharge rate and length of stay for surgical emergency GP referrals and all emergency referrals to the surgical team.

## 2 Methods

We kept a prospective spreadsheet, which was updated on daily basis, since opening of SACU in September 2016. The collected data identified all new referrals and follow-up patients attending the unit. Through this dataset we have tracked the unit's performance against our set targets and created regular reports which we utilized in our discussions to develop the unit further.

Historical data was retrieved back to April 2015 to be used as a comparison for change in practice. We identified two intervention points; the first one when SACU was opened in September 2016, the second point being the introduction of a dedicated senior surgeon in September 2017. Data up to May 2018 was included in the analysis and reported in this paper.

We looked at the effect of SACU and the dedicated senior surgeon on length of stay and same day discharge for both emergency GP and all emergency surgical referrals to the general surgical team. In addition, we looked at the effect of dedicated senior surgeon on the number of patients diverted from the surgical take to SACU and on the waiting time for senior review.

The results of this study were reported in line with the Standards for Quality Improvement Reporting Excellence (SQUIRE) criteria [11].

### 2.1 Statistical analysis

We identified three groups of patients: Pre-SACU, SACU and SACU with dedicated senior surgeon. Excel 2010 was used to create graphs and produce the descriptive statistics of our patients. To illustrate differences between the three groups, we calculated percentages, means and standard deviations; a P < 0.05 was considered statistically significant. Only new referrals to the unit were included in the analysis, any follow up patients or ward attenders were identified and excluded.

We used the one-way ANOVA test [12] to explore the effect of introducing SACU and dedicated senior surgeon on length of stay and same day discharge. If the test was statistically significant, a Tukey HSD post hoc test was performed to identify which of the groups were significantly different from each other.

Chi square test [13] was used to study the effect of a dedicated senior surgeon on the surgical take and the one tailed *t*-test [14] was used to explore if having dedicated senior surgeon in the unit had any significant effect on the waiting time to review.

## 3 Results

We identified three groups of patients: Pre-SACU (April 2015 to August 2016), SACU (September 2016 to August 2017) and SACU with dedicated senior surgeon (SACU + DSS) (September 2017 to May 2018).

In the Pre-SACU period there was a total of 4606 emergency surgical admissions, of which 52.88% (n = 2436) were GP referrals. In the SACU period there were 3219 similar admissions with 47.56% (n = 1531) GP referrals, and in the SACU + DSS period 2045 emergency admissions were seen by the surgical team with GP referrals making 37.11% (n = 759) of the total.

### 3.1 Effect on same day discharge rate

**Fig. 3:**
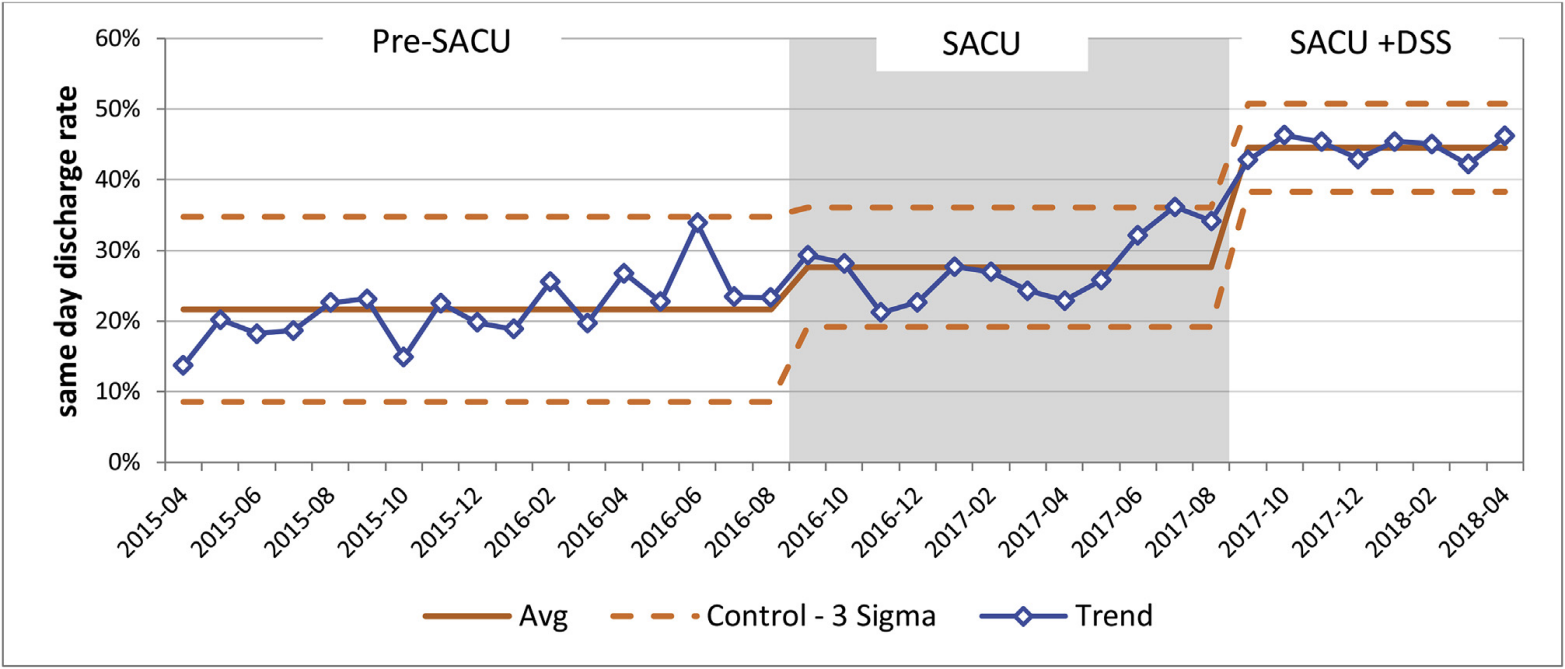
Effect of introducing SACU and dedicated senior surgeon on same day discharge for GP emergency referrals.

Pre-SACU 22% (SD = 4.81%) of emergency GP referrals were discharged the same day, this increased to 28% (SD = 4.68%) when SACU opened. This rate increased further to 45% (SD = 4.68%) in the SACU + DSS period; this is a 104.5% percentage increase compared to the same day discharge rate Pre-SACU (Fig. 3).

There was a statistically significant difference between the groups as determined by one-way ANOVA (F (2.35) = 80.88, *P* = 0.000). A Tuckey HSD post hoc test revealed that same day discharge for GP referrals was statistically significantly higher when SACU was introduced (Q statistic 4.78, *P* < 0.01). SACU + DSS group same day discharge rate was statistically significantly higher when compared with both Pre-SACU and SACU groups (Q statistic 17.86, *P* < 0.01 and Q statistic 12.6, *P* < 0.01 respectively).

**Fig. 4:**
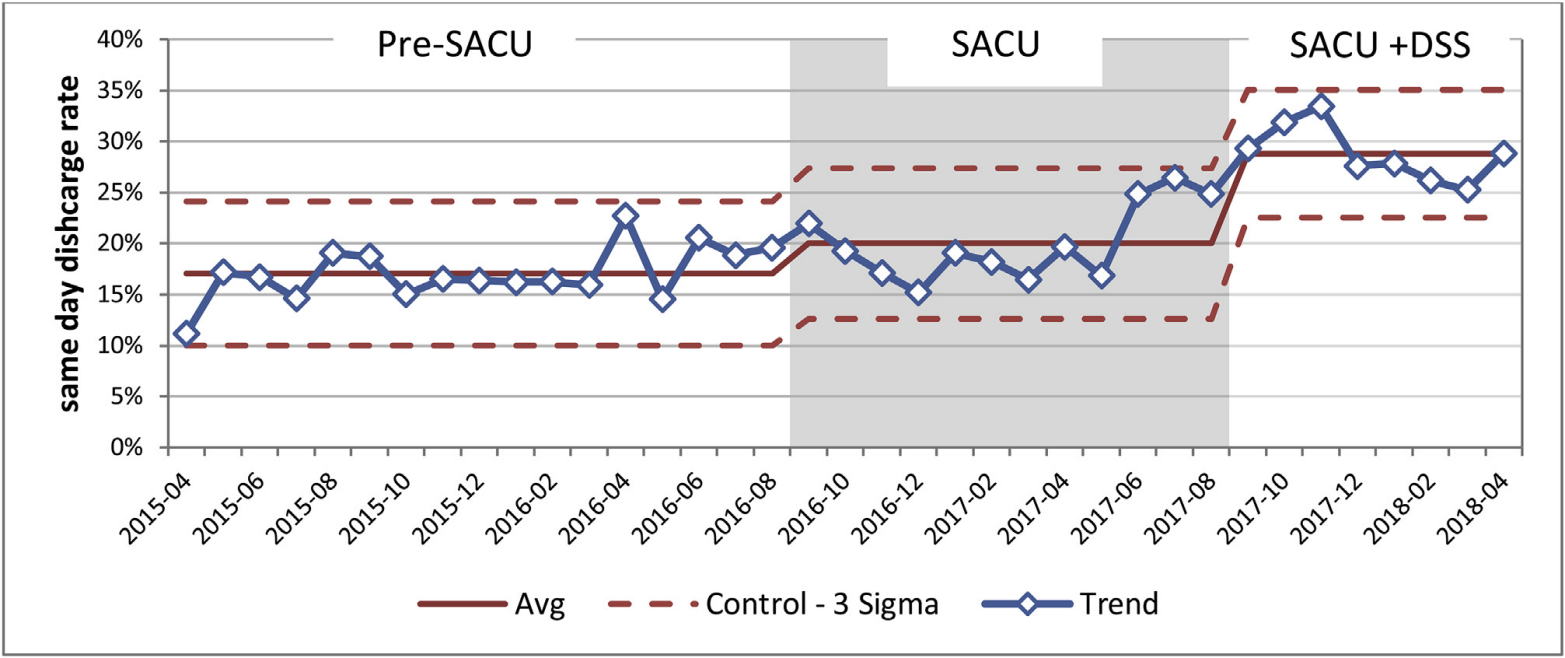
Effect of introducing SACU and dedicated senior surgeon on same day discharge for all emergency referrals.

Same day discharge rate for all emergency referrals Pre-SACU was 17% (SD = 2.68%), this increased to 20% (SD = 3.7%) when SACU was introduced. This rate increased to 29% (SD = 3.44%) when a dedicated senior surgeon was present; this is a 70.6% percentage increase when compared to the Pre-SACU period (Fig. 4).

There was a statistically significant difference between the groups as determined by one-way ANOVA (F (2.35) = 35.48, *P* = 0.000). A Tuckey HSD post hoc test revealed that same day discharge for all emergency referrals was statistically significantly higher when SACU was introduced (Q statistic 3.8, *P* < 0.05). SACU + DSS group same day discharge rate was statistically significantly higher when compared with both Pre-SACU and SACU groups (Q statistic 11.89, *P* < 0.01 and Q statistic 7.86, *P* < 0.01 respectively).

### 3.2 Effect on length of stay

**Fig. 5:**
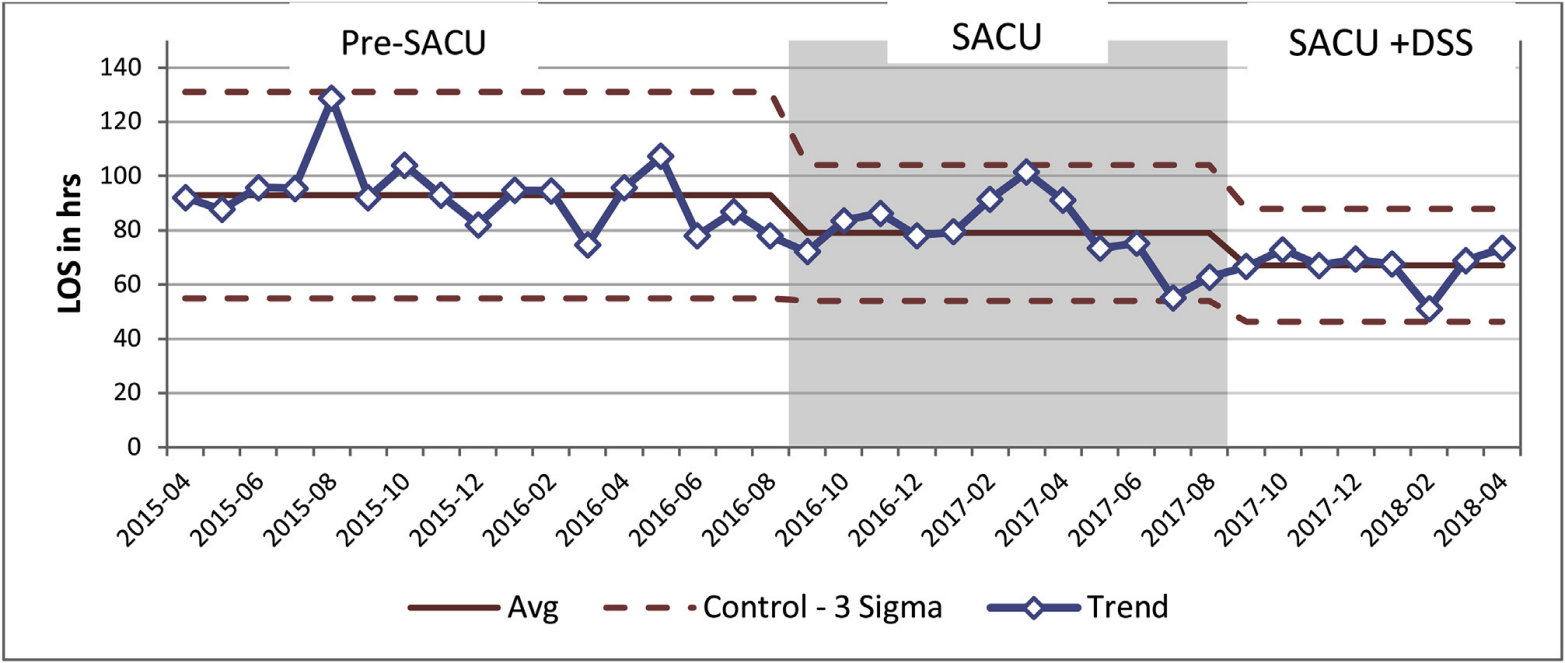
Effect of introducing SACU and dedicated senior surgeon on length of stay for GP emergency referrals.

Pre-SACU the mean length of stay (LOS) for emergency GP admissions was 92.95 h (SD = 12.75), This dropped to 79.14 h (SD = 12.8) in the SACU group. In the SACU + DSS group LOS dropped to 67.07 h (SD = 14.57), this is a 25.88 h reduction when compared to Pre-SACU group (Fig. 5).

There was a statistically significant difference between the groups as determined by one-way ANOVA (F (2.35) = 13.77, *P* = 0.000). A Tuckey HSD post hoc test revealed that LOS was statistically significantly shorter when SACU was introduced (Q statistic 3.4, *P* < 0.05). SACU + DSS group LOS was statistically significantly shorter when compared with both Pre-SACU and SACU groups (Q statistic 7.38, *P* < 0.01 and Q statistic 3.93, *P* < 0.01 respectively).

**Fig. 6:**
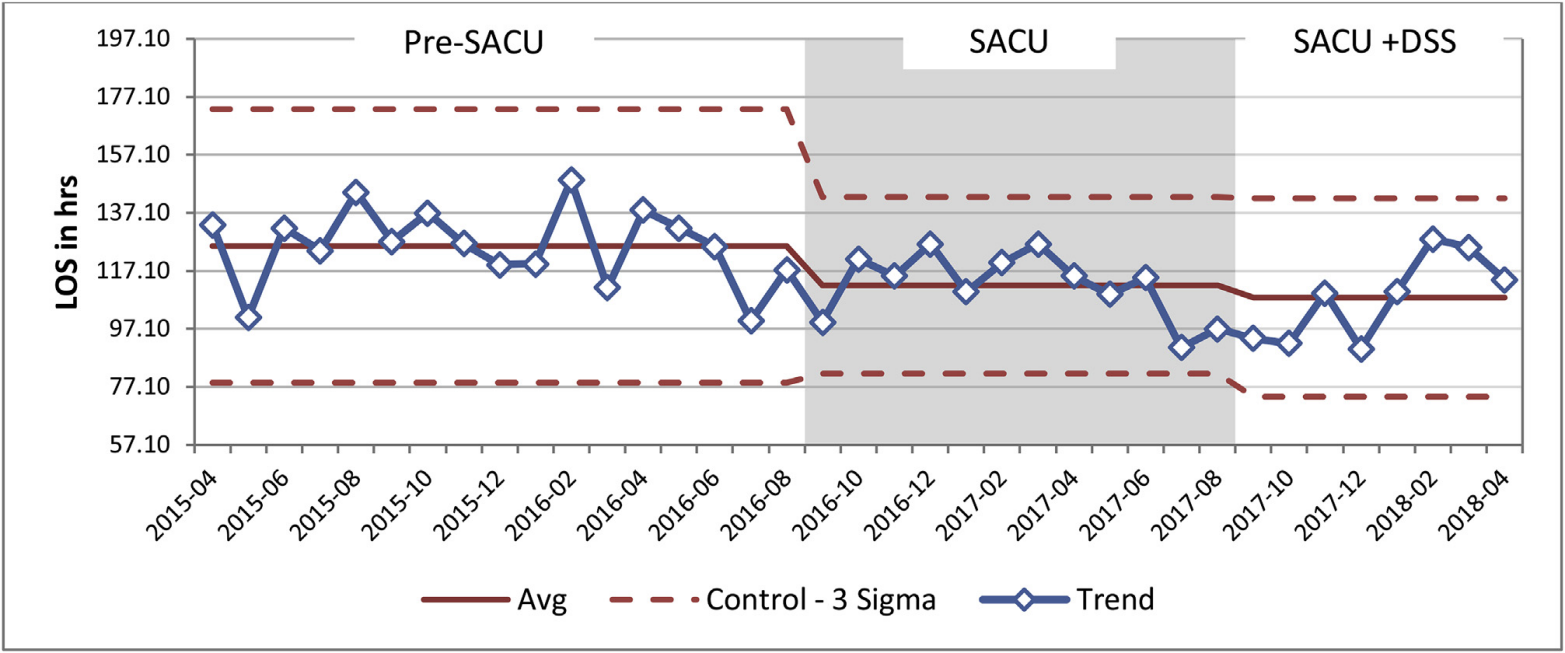
Effect of introducing SACU and dedicated senior surgeon on length of stay for all emergency referrals.

Mean LOS for all emergency admissions Pre-SACU was 125 h (SD = 13.45), this was reduced to 112.14 h (SD = 11.46) hours in the SACU group. In the SACU + DSS group the mean LOS was 107.09 h (SD = 14.55), this is a reduction of 18.6 h when compared to Pre-SACU group (Fig. 6).

There was statistically significant difference between the groups as determined by one-way ANOVA (F (2.35) = 7.51, *P* = 0.001). A Tuckey HSD post hoc test revealed that LOS was statistically significantly shorter when SACU was introduced (Q statistic 3.86, *P* < 0.05). SACU + DSS group LOS was statistically significantly shorter when compared with Pre-SACU group (Q statistic 5.06, *P* < 0.01), but there was no statistically significant difference between SACU and SACU + DSS groups (Q statistic 1.42, *P* > 0.05).

### 3.3 Effect on surgical take

**Fig. 7:**
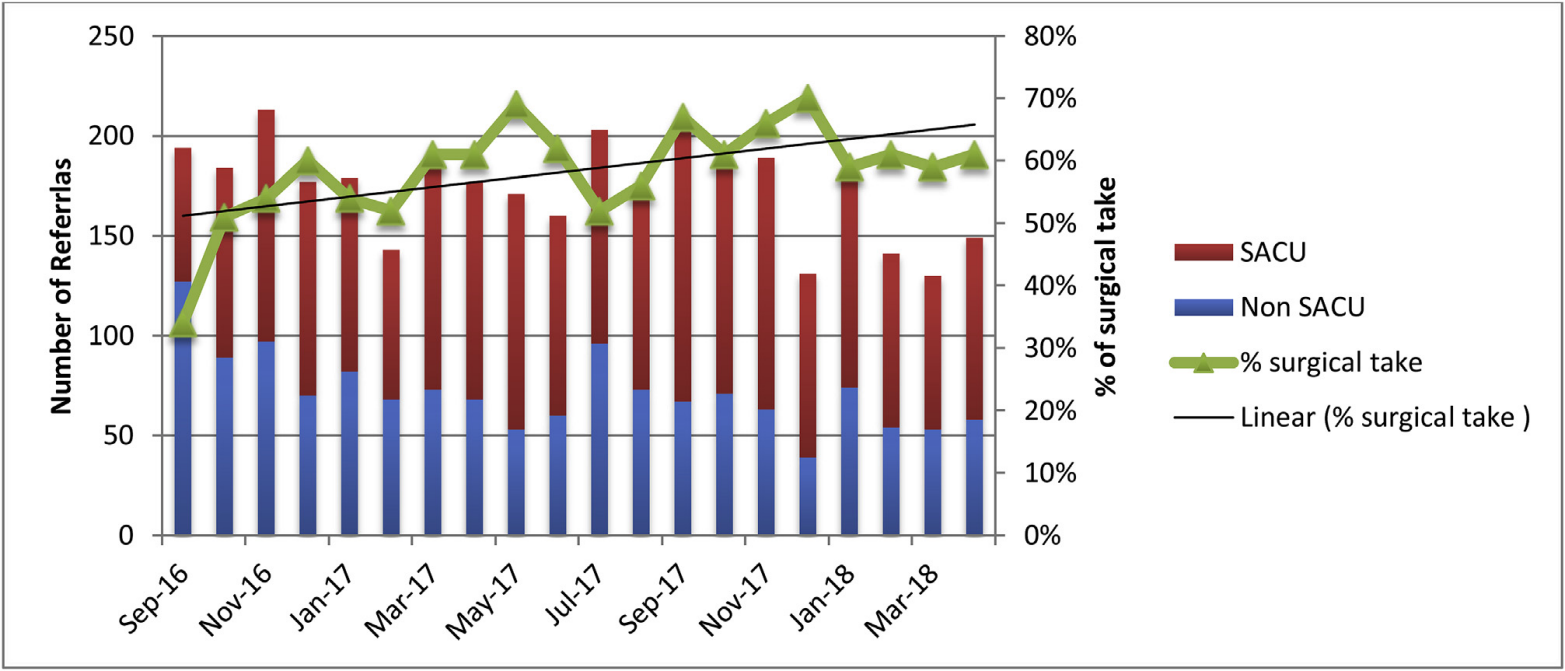
% of surgical take diverted to SACU since opening of SACU and introducing dedicated senior surgeon.

In the SACU group 55% of emergency GP referrals were seen in the unit, when compared with SACU + DSS group this has increased to 63%, a percentage increase of 14.55% (Fig. 7). Chi Square test confirmed that the difference between the two groups was statistically significant X^2^ = 20.21, *P* < 0.00001.

### 3.4 Effect on waiting time to senior review

**Fig. 8:**
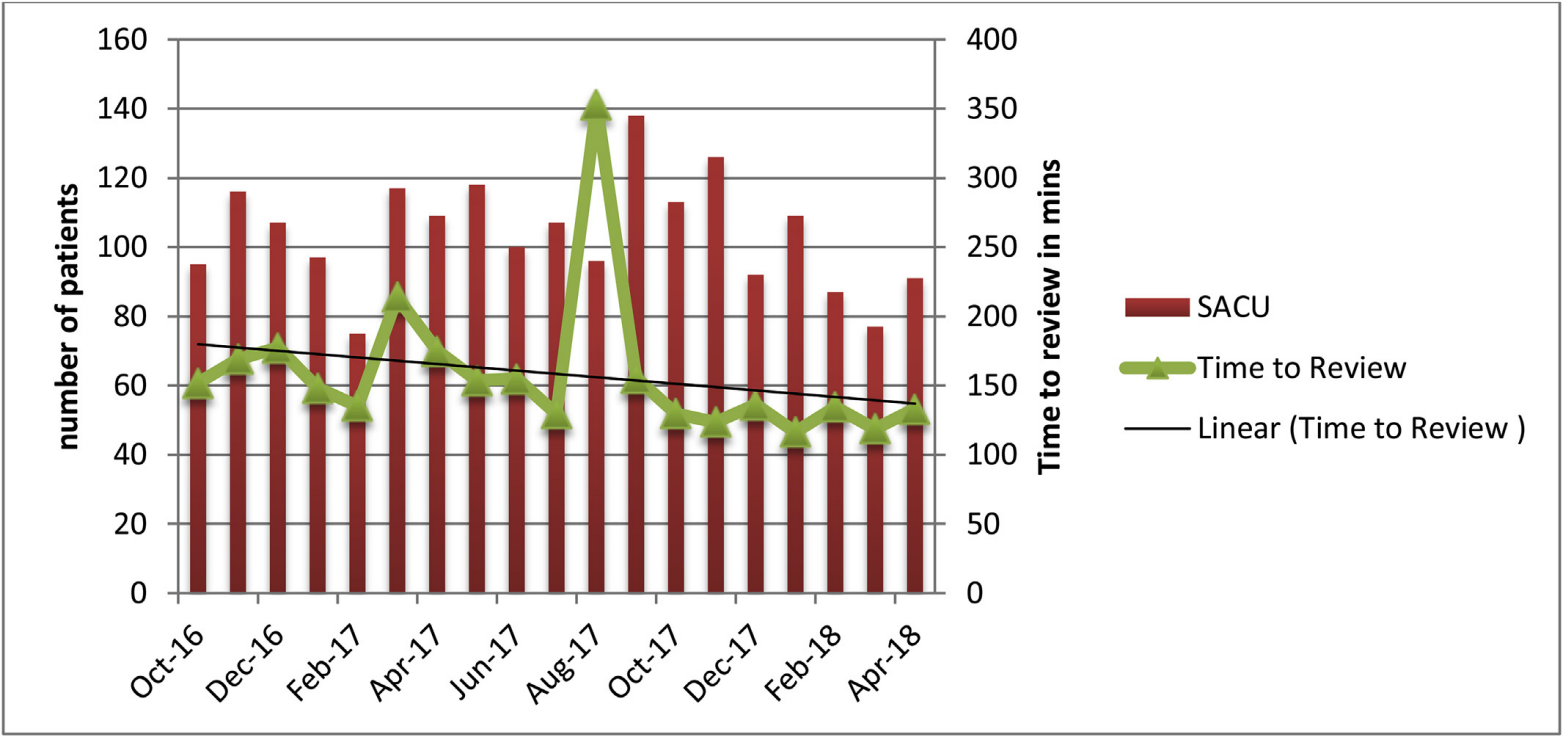
Time to senior review since opening of SACU and introducing dedicated senior surgeon.

In the SACU group the mean waiting time to senior review was 178 min (SD = 62.2), after introduction of a dedicated senior surgeon this dropped to 131 min (SD = 12.32) (Fig. 8). One tailed *t*-test showed that there was statistically significant difference between the SACU and SACU + DSS group waiting time to senior review (t = 2.1, *P* = 0.02).

## 4 Discussion

We have demonstrated that introducing a surgical ambulatory service and having a dedicated senior surgeon for the service resulted in a positive impact on length of stay and same day discharge rate in our department.

Introduction of SACU increased the same day discharge rate for emergency GP referrals to 28% and this rate was improved further to 45% by allocation of a dedicated surgeon to SACU. Although the unit only operates Monday to Friday during day time hours, there has been a clear effect on the same day discharge rate for all emergency admissions as well (70.6% percentage increase).

Bath Royal United Hospital (RUH) reported a same day discharge rate of up to 82% [10], however, this rate was only for patients attending the ambulatory unit. When we looked at our SACU unit same day discharge rate, we have managed to achieve a similar rate (80%). What we highlight in this study is the effect of SACU on the surgical take overall, and the potential for an ambulatory service to improve flow of emergency patients throughout the whole surgical service.

30-day readmission rate in our unit was 1.8% and this rate was similar to the rate (1.6%) reported by Royal Derby Hospital [8]. As the unit managed patients on an ambulatory basis, we had many patients returning for tests or procedures. In order to calculate an accurate re-admission rate correct coding of patient attendances was very important. In addition, all readmissions were reviewed on a monthly basis to see if there were any recurring trends.

The length of stay for emergency GP referrals was reduced by 25.88 h, and 18.6 h were saved from all emergency surgical admissions. This reduction has saved 102 hospital bed stays every month since the opening of SACU. This saving is similar to that achieved by equivalent units, with RUH reporting approximately 85–90 bed stays saved per month [10].

We have also noticed that introducing SACU has changed the practice within the on-call team. The service is utilized by the on-call team, who discharge patients from the emergency department to come back to SACU later for further investigations or assessment. This has contributed to the reported outcomes.

We have been actively working to pull appropriate patients into the unit, this is achieved by using our ANPs to take the referrals directly from the GPs and decide which referrals are appropriate to be seen in SACU. The dedicated senior surgeon provides a point of reference, and support for the ANPs. The success of this measure has been seen in the increase of patients diverted to SACU from the surgical take.

Providing consistent senior cover to the unit was difficult to achieve all the time. Although three surgeons agreed to include SACU in their job plan, no cross cover was agreed for when these surgeons were unavailable. This meant that the unit does not have a dedicated surgeon when one of its usual seniors is on leave or on call. On these days the on-call team is expected to provide cover, which is not an optimal situation.

The solution to the problem is to appoint surgeons with an interest in emergency surgery, who will work to develop the service further and provide consistent cover. This type of appointment has been increasing over the last few years [15], in line with increased recognition of the importance of consultant-delivered acute surgical care [16].

During winter the unit was used as an escalation area. This resulted in the unit being bedded on multiple occasions. The team on these occasions moved the unit to a different clinical area. This has helped the flow in the emergency department significantly and reduced bed pressures. However, it has raised a problem regarding patient follow up and maintaining a dedicated unit which patients can reliably contact if needed.

Unfortunately, this problem has not yet resolved, and we are still facing days when the unit is not functioning because it was bedded the night before. To prevent this from happening in the future we seek a solution based on two components. Firstly, the relocation of the unit to a clinical area which cannot physically be bedded, and secondly agreement of the executive team to stop the practice of using the unit as an escalation area.

## 5 Limitations

Limitations to the project were due to the difficulty in providing consistent senior cover for the unit. Although the ANP's were present all the time and had an important role in running the unit, provision of timely senior input was lacking on some days. The data collected on these days were included in the result as part of the SACU + DSS group, this was responsible for some outlying points within the data. Having a clear plan for providing consistent senior cover is important if this study is to be implemented in other units.

## 6 Conclusion

Establishing an emergency surgical ambulatory service has reduced length of stay and saved significant hospital beds stay by increasing the same day discharge. This effect was enhanced by having a dedicated senior surgeon providing early input and decision making.

These units will not be able to produce the intended effect without a dedicated team, easy access to diagnostic tests, location in a protected clinical area and the support of the senior management team in the hospital.

## Conflicts of interest

None.

## Sources of funding

None.

## Ethical approval

The project was discussed with the research and development lead in the trust and decision was taken that this project does not require application for ethical approval as it is a quality improvement project and will only report on local outcomes.

## Research registration unique identifying number (UIN)

researchregistry4613.

## Trial registry number

N/A.

## Author contribution

**Mohammed Ali Kazem**: Conceptualization, Methodology, Validation, Formal analysis, Investigation, Writing - original draft, Visualization.

**Claire Hopley**: Conceptualization, Methodology, Software, Resources, Data Curation, Writing – Review & Editing, Project Administration.

**David Corless**: Investigation, Writing – Review & Editing, Supervision.

## Guarantor

Mohammed Ali Kazem.

## Provenance and peer review

Not commissioned, externally peer reviewed.
